# Praxeological analysis (PA/CPA) for stigma, health inequalities, and coercion in women’s services

**DOI:** 10.3389/fpsyt.2026.1766029

**Published:** 2026-02-16

**Authors:** Phil Hutchinson, Loreen Chikwira

**Affiliations:** 1 School of Psychology, Manchester Metropolitan University, Manchester, United Kingdom; 2 The King’s Fund, London, United Kingdom

**Keywords:** Critical Praxeological Analysis (CPA), Praxeological Analysis (PA), trauma-informed care, women’s mental health, restrictive practices, coercion and restraint, stigma in healthcare, culture of care

## Abstract

This paper sets out a practical, interview-free protocol for studying how stigma, inequalities, and restrictive practices are done in women’s mental health, learning-disability, and autism services, with a particular focus on women detained in inpatient and other institutional settings. Building on Praxeological Analysis (PA) and Critical Praxeological Analysis (CPA), we specify data pathways for naturally occurring materials (clinical letters, triage logs, ward round notes, safeguarding records, complaint correspondence, public hearings/transcripts, and, where ethically approved, audio/video recordings of clinical interactions between people who use services and healthcare professionals), and a replicable analytic procedure keyed to linguistic/praxeological Gestalts. Rather than treating “stigma” as an attitude, attribute or variable, we investigate stigma in its sites of production, the situations in which discrimination, discreditation, degradation etc are done, experienced and witnessable. The protocol operationalizes what we call a praxeological respecification: a shift from traits to scenes and from beliefs and attitudes to practices, enabling research and quality improvement that directly addresses the levers through which inequalities are produced and maintained and which supports least-restrictive, trauma-informed, autism-informed and culturally competent inpatient care in line with current NHS culture-of-care standards.

## 1 Rationale and positioning

Work to reduce restrictive practices and address stigma in women’s health and care services is often framed in terms of policy commitments, training, and values (1). Those matter. But whether care is least-restrictive and non-stigmatizing is also decided in the small: in how concerns are heard, how risk and responsibility are formulated, how refusals are treated, and how the record sets up the next encounter. This paper offers a practical protocol for making those mechanisms visible in real service material and for turning analysis into concrete alternatives that teams can use.

### 1.1 What this protocol is for

The protocol is designed to help teams see, case by case, how restriction and stigma can become live options in routine clinical scenes—and how they can be avoided without losing sight of safety. It does this by working with naturally occurring service materials (records, letters, call logs, MDT/ward-round documentation, safeguarding forms, complaint correspondence, and where feasible recordings/transcripts) and by treating them as evidence of how a case was heard, handled, and carried forward.

The protocol is service-facing because each analysis is designed to yield two practical outputs. First, it specifies one or two alternative next moves: concrete things a clinician could say or do that still keep safety in view while also showing that the person has been heard. Second, it offers a documentation rewrite: a better version of the line that will prefigure the next encounter. These two outputs—a short list of alternative next moves and an example of better documentation—are what make the method usable in supervision, debriefs, audit and coaching, and service reviews. For example, instead of “Refused observations; firm boundaries required”, a reworked note might read “Discomfort with visual checks; proximity checks agreed; review at 15:00”, which keeps safety in view without casting the person as non-compliant.

### 1.2 Alignment with culture-of-care standards

NHS England’s culture of care standards (2) for mental health inpatient services ask providers to deliver least-restrictive, trauma-informed, autism-informed and culturally competent care, with a strong focus on relational safety, equality, and avoiding harm. Our protocol offers a concrete way to do this analytic and improvement work: it shows, case by case, how women’s concerns are or are not heard; how decisions about leave, observations, seclusion, escalation or medication are made reasonable in the moment; and how those decisions are written into the record in ways that either reproduce or interrupt stigma and inequality. In that sense, PA/CPA provide a practical tool to support culture-of-care ambitions at ward level, rather than an additional framework for staff to “hold in mind.”

### 1.3 Conceptual resources and stance

Conceptually, the protocol draws on the philosophical work of Ludwig Wittgenstein (3) and Frank Ebersole (4), and the ethnomethodology and conversation analysis (EM/CA) of Harold Garfinkel (5) and Harvey Sacks (6, 7). From Wittgenstein and Ebersole we take an orientation to meaning-in-use: there is no freestanding content for a term outside the organized scene in which it figures; an investigation oriented to the meaning-in-use in human situations is called a *grammatical investigation* (GI) (8, 9). From Garfinkel and Sacks we adopt deference to methods displayed by members through which the situation’s order and sense are produced, and to the idea that structure is recoverable in the particulars of a case. Read together, this commits us to recover what a bit of talk or writing is doing here before we draw any lessons from the words it uses. That grounding lets us show how “stigma” is produced in interaction, the work the term does when invoked, and the way in which *stigma* often functions as a category term and gloss across diverse experiences (e.g., being demeaned, discredited, excluded, stereotyped, marginalized), rather than as the name of an inner attitude, attribute, or discrete “thing.” By category term we mean a word that gathers together a family of different experiences (much as the term *emotion* gathers shame, anger, fear, grief, and so on). By gloss we mean an occasioned summary expression—used by participants, practitioners, and analysts—that *packages* a complex, morally consequential scene without settling *what, exactly, happened* or *how it was done*. In ordinary use, saying “she was stigmatized” typically makes relevant a next question such as “In what way?” or “What happened?”—and that is the analytic move we make: we return from the gloss to the situated practices through which something becomes intelligible (or contestable) as stigmatizing in that setting (10, 11). These commitments also justify beginning from single cases. If structure is recoverable in particulars, a perspicuously presented case can warrant an analysis; neighboring cases are then selected to test, refine, and qualify it—without turning the exercise into induction from a “sample” or into a search for portable indicators to be applied by template.

These conceptual commitments matter because they keep the analysis answerable to the scene and direct attention to where services can intervene: the next move in the interaction and the line that enters the record. That is why the protocol is designed to end, in every case, with the two practical outputs set out in Section 1.1: alternative next moves and a documentation rewrite.

### 1.4 Meaning-in-use principle

Throughout, we observe the meaning-in-use principle. We do not treat words or labels as if they carried a fixed meaning around with them. Many studies of stigma are organized around identifying terms that are taken to be experienced as negative (for example, “non-compliant” or “frequent attender”) and then examining patterns of their use, or asking how such labels are received and felt. Our approach is different. We are interested in what a word or phrase is doing in a particular moment of talk or writing: which activity it belongs to, where it comes in the sequence, and what it makes reasonable to do next. The same expression can do different work in different scenes. A label may, in one context, help organize care; in another, it may quietly downgrade credibility or make restriction look like the only sensible course. Meaning, for our purposes, is therefore use in its practical context, not a freestanding property of terms. For a fuller statement of this stance see Hardman and Hutchinson (12), Diskin and Hutchinson (13), and Hutchinson (11).

The meaning-in-use principle matters for stigma and restrictive practice because it shifts the focus from “bad words” or “negative attitudes” to the local work that talk and documentation are doing. Rather than coding terms as inherently stigmatizing, we show how particular formulations, in particular slots, narrow or widen the live options and how lines in the record carry those options forward to the next encounter. That is where the practical levers are: in the way a case is formulated, responded to and written up, turn by turn. The rest of the paper sets out the data pathways, ethics, analytic steps and reliability checks that allow us to do this systematically.

## 2 How moves make possibilities live (GI → EM/CA → PA/CPA)

Drawing on EM/CA, PA and CPA provide a way to show—case by case—how particular moves in talk and action make some possibilities live and render others latent or moot. It is speakers and actors who accomplish this, moment by moment, by how they place their turns and actions in relation to one another. Seen through an EM/CA lens, we can describe this as sequential organization: turns are designed so that they make some next actions relevant (prospective) and display how prior turns were understood (retrospective). Our analyses, therefore, track how a move *practically* establishes local norms and expectancies and how subsequent conduct either fulfils, resists, or repairs those expectations. We do not ascribe meanings to words in the abstract; we show what a formulation does here, in this slot, and what that does to the range of reasonable next steps.

### 2.1 Adjacency pairs

In EM/CA, adjacency pairs are familiar two-part sequences such as greeting–greeting, question–answer, offer–acceptance/refusal, request–grant/decline. They matter for our purposes because they show—in a very local and inspectable way—how interaction is *action-guiding*. A first action makes a limited range of second actions relevantly expected. That “expectability” is not a rule imposed from outside; it is something speakers display that they are oriented to, and they manage it through what they do next (14, 15).

Consider a greeting or a question. A greeting makes a return-greeting relevant; absence of it typically invites repair—”Hello?”—or an account, and, until repaired, it casts the encounter as strained. Likewise, a question makes an answer relevant; a non-answer (e.g., “Well, that depends…”) establishes a different project (negotiation, reframing, resistance) and requires the next speaker either to accept that re-keying or to pursue an answer, with implications for alignment. These are not dictionary facts about “greeting” or “question”; they are local norms made operative by position in the sequence. When introducing adjacency pairs in his *Lectures on Conversation*, Sacks depicted the normative work succinctly: “Various sorts of things come in pairs, specifically in adjacent pairs, such that if a first is done to somebody, then the somebody to whom the first is done does a second in return” (6).

It is important here to keep in mind our meaning-in-use principle. The same utterance can serve different roles and thereby be the first part in different adjacency pairs, dependent on use. In an opening in a clinical setting “How are you?” might operate as question which invites an answer as its second part, whereas the same utterance in corridor talk is likely to be phatic and is operating as the first part of a greeting pair (6). The same words can belong to different Gestalts, be doing different work, and so invite or deter different next moves. The general point illustrated by adjacency pairs is the extent to which conversations, and interaction more generally, serve as the production site of normativity. That is to say, as conversational turns unfold, what is relevant, saliant, expected and reasonable is ongoingly produced, maintained and repaired. In asking a question, a speaker invites an answer as the next turn.

There is extensive, and detailed work on this feature of conversation, dating back to Sacks’ own earliest lectures, delivered in the early 1960s, but for our purposes here, we want to understand how certain possibilities, such as physical restraint, might be rendered saliant in the interaction, as the interaction unfolds. What kind of interactional or conversational sequence might put physical restraint on the table, so to speak, as a reasonable course of action? And how, once this becomes a course of action that is now locally reasonable, is that experienced by the patient and made witnessable in their responses? Adjacency pairs therefore provide a simple window onto how interaction is action-guiding: first moves make particular kinds of second moves relevantly expectable, and departures are managed through pursuit, accounts, and repair. This is the basic machinery we later draw on to show how, in clinical sequences, certain options can come to look like the reasonable next step.

### 2.2 Membership categories in use

Membership categorization is a way of seeing how talk organizes persons and actions by drawing on categories (e.g. “mother”, “autistic person”, “frequent attender”) and the membership categorization devices they belong to (such as family, clinic, service-user) (16–18). The analytic focus is not on what people “are” but on how, in conversation, these categories are invoked, made relevant and serve an organizational role. Categories come with category-bound predicates and activities—expectations about what kinds of troubles, responsibilities and remedies “go with” that sort of person—and so their use introduces local norms or rules into the scene. Labels such as *woman*, *mother*, *autistic*, *learning-disabled* or *frequent attender* do not carry freestanding meanings; in use, they organize what counts as relevant trouble, responsibility and remedy in a scene and orient co-participants to a way of seeing what is going on and to a range of next actions that now look natural, necessary, or ruled out.

For example, writing “frequent attender” in a file entry does not simply record a fact about visit frequency. In many services it functions as a boundary-relevant category: it makes it reasonable to ask whether the service is being over-used, to discuss limits, or to shift attention from the person’s present difficulty to their history of contact. In that sense it projects boundary-work (gatekeeping, rationing) as the next activity and tends to sideline credibility-first work on what the person is now reporting. By contrast, a line such as “reports pain worsening since last week; seeks plan for sleep” makes problem-solving and planning the relevant next actions. The label and the alternative formulation belong to what we call different *praxeological Gestalts*: each brings a different pattern of expectations, questions and justifications into view.

Something similar can be seen with formulations like “capacity as a mother remains a concern,” “lacks insight,” or “non-engaging.” These phrases do not operate as neutral summaries of an assessment; in practice they often pre-figure escalation, increased surveillance or withdrawal of options. They invite readers to treat subsequent resistance, hesitation or disagreement as further evidence of the very category invoked. In contrast, category-light descriptions that foreground situational particulars (for example, clashes of appointments, sensory overload, availability of support at home) tend to project support-planning and negotiation as next steps. In all of these cases, the category work is visible not in the word taken in isolation but in what the formulation makes reasonable to do next, and in how the line will travel to shape the next encounter.

### 2.3 Gestalts

We use “Gestalt” to pick out a meaning-relational whole: a pattern of activity in which each move draws its sense from its place in the whole, while reciprocally serving to produce the identity of the whole. In PA/CPA this is a praxeological or linguistic Gestalt (12, 13)—not an inner mental structure, but the organized scene itself (a clinic consultation, a safeguarding review, a ward round, a queue at reception) as participants live and produce it. Gestalts matter here because we are interested in how particular formulations and actions belong to, and help to constitute, the local setting in which restrictive practices (such as cancelled leave, increased observations, seclusion, segregation or rapid tranquillization) can come to seem natural, or even necessary, or, conversely, in which such measures are treated as out of place and alternatives are actively pursued. In PA/CPA we treat each move as a member or constituent of a praxeological (linguistic) Gestalt—a meaning-relational whole produced in and through the activity. A move does not only project a range of reasonable or relevant next turns-at-talk or actions; it also helps constitute the whole scene that confers sense on its parts. The normative force here is best understood as guidance rather than hard constraint: a greeting, for example, orients the next speaker toward a return-greeting, but departures are possible and, if they occur, they call for repair or accounting.

### 2.4 Same words, different Gestalt

Consider again the example we introduced briefly, above (2.1): “Hi, how are you?” uttered by a doctor. In the consulting room, during the morning’s clinic, as an opening, it functions as a clinical question—it makes a medically relevant report the next reasonable move, and a non-answer invites pursuit or reframing for care-planning. By contrast, the same doctor, leaving for home, passes the same patient now stood by a vending machine in the hospital foyer and says, “Hi, how are you?” Here it functions as a greeting: a return-greeting or a brief phatic response is the locally expected next move. Offering a detailed symptom report in this setting would be out of place unless one or both parties do additional work to shift what is going on—for example, the doctor stops, signals a move back into a clinical encounter (“Let’s talk about that properly—shall we book you back in?”), or the patient explicitly treats the exchange as an opportunity to reopen the consultation. The same words establish different normative guidance for next-turn possibilities because the Gestalt—activity, role-relations, time and place—differs (6).

### 2.5 Discordant Gestalts (CPA)

In CPA we also make use of the notion of *discordant Gestalts* to capture cases where participants are not, in fact, inhabiting the same praxeological whole. Here, the trouble is not simply that one party “doesn’t understand” or has breached the local norms, but that different patterns of salience, relevance and obligation are in play at once. A clinician may be oriented to a risk-management Gestalt in which past self-harm and missed appointments make escalation and restriction look salient and necessary; the service-user may be oriented to a survival or care-seeking Gestalt in which the same actions express endurance, distrust or the search for a liveable arrangement (10, 13, 19). The same words, categories and records can then be heard and used differently, producing friction, misrecognition and, often, a sense on the clinician’s side that the patient is “non-engaging” or “lacking insight”, and on the patient’s side that they are being stigmatized or written off. In stigma cases, CPA treats such moments as sites of discordant Gestalts, in which the conditions under which restraint or exclusion become “reasonable” for one party are precisely those under which the other experiences degradation, discreditation or being placed outside the circle of the “fully” accountable.

### 2.6 Two short clinical illustrations

Taken together, these points about sequential organization, category work, praxeological Gestalts and discordant Gestalts can look abstract. To make them concrete, we now turn to two short clinical illustrations. The first shows how sequential norms around proposal and refusal can re-key a patient’s stance into a risk display, making restriction a live and then expected option. The second shows how the local invocation of a membership category brings with it a stock of category-bound predicates and inferences, so that boundary-talk and limit-setting become the reasonable next actions under the heading of “stigma”.

#### 2.6.1 How refusal becomes a live warrant for restriction: the sequential production of norms

On an acute ward, a doctor proposes “Q15 observations” [checks every 15 minutes]. The patient says, “I don’t want people staring at me.” If the next move pursues the proposal (“Refusing observations increases risk”) rather than offering a safety-equivalent alternative (e.g. proximity checks, patient-initiated call-button, a timed review), the refusal is recast as a risk display. That re-keying then makes “cancel leave” appear reasonable; and when the documentation later reads “Refused observations; firm boundaries required”, the record carries this reading forward so that, at the next contact, restriction is already live and perhaps even expected. A different next move (name an alternative; trial; schedule a review) keeps non-restrictive options live and writes a line that travels differently: “Discomfort with visual checks; alternative proximity checks agreed; review at 15:00.”

#### 2.6.2 How a category invocation might make boundary-talk live: stigma in practice

At triage, the clinician looks up the file and says, “I see you were here last week … we have a lot of frequent attenders.” In this slot, the label does not report a neutral fact; in use it invokes a membership category and mobilizes a familiar set of category-bound predicates and inferences (service over-use, time-wasting, need for limits). It therefore projects boundary-work as the next reasonable activity and tends to downgrade the caller’s credibility. By contrast, starting with a credibility-first formulation—”You’re frightened it’s worse today since the baby; we can safety-plan now or book a perinatal slot for first thing”—projects planning as the next activity and keeps restriction off the table. The record then travels differently: “Frequent attender” makes limit-setting live next time; “Safety-plan agreed; perinatal review 09:00” makes care-planning live.

The method that we lay-out in what follows is simply the disciplined recovery of these praxeological Gestalts in short passages—showing how particular moves project some next actions, dim others, and write records that carry those possibilities forward.

## 3 Data sources & pathways

### 3.1 Corpus construction (qualitative case-selection and curation)

We treat corpus construction as a qualitative case-selection principle: assembling a small, curated set of passages drawn from recognizable activities (e.g., triage, ward round, safeguarding review) and—where possible—linking modalities (recordings/transcripts, notes, minutes, complaint correspondence) so we can track how a formulation is used in the scene and then how the resulting line “travels” into the record and into later encounters. The aim is not representativeness by frequency, but *comparability of use in context*: cases are selected because they allow us to inspect how particular formulations, category invocations, proposals/refusals, and risk talk are being handled *in situ*.

Crucially, “neighboring cases” need not be repetitions of the same pattern. They may be comparative (similar sequential position but different handling), contrastive (the “same words” doing different work in different activities), or deviant (a case that resists the emerging reading and forces revision). In this way, a corpus can be built to show both (i) recurring ways restrictive options become reasonable next, and (ii) the alternative ways the same practical terrain can be organized so that non-restrictive options remain live. This approach is consistent with practical guidance on working with qualitative materials across text, image, and sound (20), but it is tailored to PA/CPA’s scene-first analytic aims.

### 3.2 How to assemble a small corpus

Choose five to ten short passages (8–20 lines) from activities you can name—an assessment, a handover, discharge planning, a safeguarding discussion. Favor extracts where a decision is being shaped or a plan is being negotiated. Keep a simple log (date, setting, type of activity, where stored). The aim is not size but clarity of scene. For trusts already working with tools such as the Patient and Carer Race Equality Framework (PCREF) (21), restraint inequality audits and the new culture-of-care standards, this kind of small, well-specified corpus provides concrete cases through which to understand *how* inequalities and restrictive practices are being reproduced locally and where alternative paths are available.

### 3.3 Storage and governance in brief

Public materials: redact identifiers and store securely. Internal documents and recordings: follow a local service-evaluation or research route, restrict access by role, and record your redaction choices. In all cases, preserve the words needed to see the action; remove only what is not required to recognize the scene.

## 4 Ethics & governance

Two distinctions keep ethics straightforward. First, distinguish public-domain from internal materials. Public items typically need only careful redaction and secure storage; internal items require local approvals and a brief data-protection note. Second, distinguish documents from recordings. For recordings, presume capacity unless assessed otherwise, use process consent (seeking and re-seeking agreement before, during and after), offer accessible information where appropriate, and adopt a disruption stop-rule so that recording is paused or abandoned if it interferes with care. Recent healthcare CA studies show this is workable in practice in sensitive settings, including end-of-life care (22).

## 5 Analytic framework (PA/CPA)

In this section, we set out the analytic protocol in three linked parts. Section 5.1 lists the *questions* we ask of any passage (scene, sequence, category/risk work, documentation travel). Section 5.2 then describes the *procedure*—how we work through the passage step-by-step, extending it where needed to avoid decontextualized readings. Section 5.3 states the *checks* that keep claims accountable (what the subsequent turns display, stability when adjacent turns are added, and brief stakeholder data sessions where appropriate). Readers unfamiliar with EM/CA terminology can treat this as a practical workflow: identify the scene, follow what the turns make relevant next, note what gets written into the record, and then produce two concrete outputs (alternative next move and documentation rewrite).

### 5.1 What we ask of every passage

1. What is the scene?

Name the activity and the shared task (e.g. assessment, handover, discharge planning, safeguarding review). What are staff, the woman, and (where relevant) her family or support network actually trying to do here?

2. What would count as showing you have heard the person?

Given what the person has just said or done, identify the next turn that would reasonably show understanding—for example, reflecting back their concern, offering a safety-equivalent alternative, or asking a clarifying question—rather than immediately correcting, dismissing, or escalating.

3. What are labels and risk talk being made to do here?

Track how categories and risk formulations (e.g. “frequent attender”, “non-engaging”, “high risk”) are brought into the scene and what practical inferences they enable—what they make it seem natural to do next, and how they influence the line that will go into the record.

### 5.2 Working through the passage step-by-step

- *Set the scene*. In one or two sentences, say where we are, who is involved, and what they are trying to do at this point (for example, “nurse and patient on an acute ward, agreeing an observations plan for the evening”). This gives enough context for the talk to be recognizable.
- *Lay out only what is needed*. Select a short stretch of talk or text (usually 8–20 lines) that shows how one move leads to another. We include just enough to see the sequence of proposal, response, and follow-up.
- *Track formulation-in-use*. Mark where labels and risk formulations appear (for example, “non-engaging”, “frequent attender”, “high risk”) and note what they do in that slot—what they make it seem natural to do next.
- *Look ahead to the record*. Note what actually gets written into the notes, or what is most likely to be written, and how that wording will shape the next contact (for example, whether it will make restriction look already justified).
- *Formulate alternatives*. Spell out one or two specific next moves that would have shown the person they had been heard while still keeping safety in view and offer a better wording for the documentation line.

### 5.3 Where warrant comes from

We treat the data themselves as the main source of evidence. That means looking at how other people respond in the interaction (what happens immediately afterwards, and how things are picked up a little later) and at what is actually written in the notes. Our reading of a passage should still make sense if we add a few lines before or after, or if we bring in a neighboring case; if the wider material shows that the situation was actually organized differently, we say so.

We also recommend brief data sessions, organized as a deliberative forum in which analysts, practitioners, and people with lived experience work through the same extract together. The point is to make the reading something others can see, question, and improve: to set out and compare different plausible ways of understanding the extract, to check them against what participants show in what happens next (and how it is taken up a little later) and what is written into the record, and to agree what can responsibly be claimed from the material.

We start from single cases because, following Sacks, a great deal about local norms and structures can be seen in the details of one encounter. We then add a small set of neighboring cases to show a family resemblance—not to produce statistics, but to make a pattern visible. This is in line with work on case studies, where concrete, context-dependent knowledge can be expert knowledge, and strategically chosen cases can test and illuminate general claims (23, 24).

### 5.4 An example (showing 5.1–5.3 in action)

A clinician proposes increased observations overnight. The patient says she can’t sleep with constant checks and asks for a different arrangement. A second staff member adds, “non-engagement has been a pattern,” and the proposal is pursued as a safety requirement. The record later reads: “Refused observations; firm boundaries required; consider escalation.”

Scene and task (5.1).

The activity is not “conversation in general” but an institutional task: balancing safety planning with rest and dignity in a perinatal inpatient setting.

What would count as showing hearing (next move) (5.1).

At the patient’s request for a different arrangement, a hearing move would treat the stance as workable: offer a safety-equivalent alternative (e.g., proximity checks, timed review, patient-initiated call-button), rather than treating the request as refusal-as-risk.

Category/risk work (5.1).

“Non-engagement” is not a neutral description here; in this slot it supports a practical inference: the patient’s grounds need not be negotiated, and boundary-talk becomes the relevant next activity.

Record-travel (5.1).

“Refused observations; firm boundaries required” does future work: it pre-formats the next encounter so restriction is already live and increasingly expected.

Step-by-step working through the passage (5.2).

Extending the extract by a few turns shows whether alternatives were offered, whether the patient accepted a partial plan, and whether staff pursued clarification or moved directly to escalation. This extension often changes what it is responsible to claim.

Accountability checks (5.3).

We test our reading against what happens next (does the team pursue compliance talk, does the patient account again, is there a later complaint referencing being “treated like a risk”)?. We also check stability under extension: if later turns show staff *did* offer alternatives and documented grounds, then “firm boundaries” may function differently. The two outputs remain the same type, but they become better targeted.

#### 5.4.1 Outputs

Alternative next move:

“I hear that checks every quarter hour will stop you sleeping. We can meet the safety aim by (a) proximity checks without waking you unless needed; (b) a timed review in two hours; (c) you can use the call-button if you feel worse. Which is workable tonight?”

Documentation rewrite:

“Patient reported sleep disruption with frequent checks; agreed safety-equivalent plan (proximity checks + review time + call-button). Review at 02:00; escalation criteria specified.”

## 6 The CPA audit & coaching tool

In practice, teams need two aids: a short map of high-risk formulations and sequence patterns, and a seven-question prompt that anchors supervision and incident reviews.

### 6.1 Formulation-in-use

We do not list “trigger terms” or assign meanings to phrases in the abstract. What matters is how a formulation lives in a scene. Three compact examples show the point.

1. “Won’t engage,” post-first refusal (triage). In a first contact, a caller hesitates, asks for time, or declines an option. Writing “won’t engage” at this moment re-keys a single, repairable stance into a trait and forecloses repair. What belongs is a re-doable next move: spell out two or three alternatives the caller can accept now, and record which was chosen. The sense of “won’t engage” here is the work it does to close-off options as the interaction unfolds, not a dictionary meaning or neutral description.
2. “Frequent attender,” before concern-first formulation (urgent care). When history is invoked before the person’s present concern is formulated, “frequent attender” tends to reframe help-seeking as misuse and to invite boundary-talk rather than planning. In cases where a credibility-first line is written first, later notes show better alignment. Again, the term’s sense is to be found in its use in this sequential slot. We are interested in the work the category term is doing here and the norms initiated which invite certain next actions and deter others.
3. “Refused observations,” without grounds and alternatives (inpatient). A bare record of refusal turns a safety negotiation into a risk flag. A better line documents the person’s grounds, the safety-equivalent alternatives offered, and the agreed review. The contrast is not between two dictionary entries but between the different work terms can do and how this directs the unfolding interaction.

Across such cases, our target is not vocabulary but formulation-in-use: what this turn does here and how the line entered in the record will shape the next encounter.

### 6.2 Seven coaching questions and embedding in routine practice

1. What scene is this (and what shared project is in play)?
2. Given the last turn, what next move would have shown understanding—and was it offered?
3. When the person said “no” or held back, was this treated as a *repairable stance*—something to talk about, clarify and offer alternatives around—or was it treated immediately as a *risk cue* that justified moving to restriction (for example, cancelling leave or increasing observations)?
4. Which membership categories (e.g., woman/mother/autistic/LD/PD) were invoked, and what *practical inferences* did they enable?
5. How did risk talk function here—did it close options or organize collaborative alternatives?
6. What will the record say, and how will that shape future encounters?
7. What specific alternative next moves could be tried next time?

Here we treat refusals and hesitations as stance displays: they show how the person sees things at that moment and can often be worked with (for example, by offering safety-equivalent alternatives), rather than being treated straight away as evidence that risk has increased.

#### 6.2.1 Embedding in practice

These prompts are intended to be used in everyday work, not just in research projects. They can be built into supervision, MDT reviews, debriefs and perinatal meetings. Teams can then track a few simple descriptive indicators (for example, complaints upheld, restrictive events, frequency of “non-compliance” codes) and compare them with reworked documentation lines, to see whether changes in how cases are formulated are linked to changes in how often restrictive options are used.

### 6.3 Ethical governance and recording pathway

Ethical governance is straightforward if we keep two distinctions in view. First, distinguish public-domain from internal materials. The former require redaction and secure storage; the latter additionally require local approvals and a short account of data protection measures. Second, distinguish documents from recordings. For recordings, assume capacity unless assessed otherwise, adopt process consent (seek, and re-seek, consent during and after the encounter), provide accessible participant information where appropriate, and include a disruption stop-rule so that recording is paused or abandoned if it degrades care. These safeguards sit comfortably with an interview-free protocol and allow small, careful collections to be assembled in routine services.

- Decision tree distinguishing public-domain sources (minimal risk) from service-evaluation/internal clinical materials (DPIA, consent/waiver, anonymization).
- Redaction protocol: roles not names; blur locations; preserve *actionable phrases* verbatim; paraphrase peripheral content.
- Handling sensitive categories (violence, pregnancy, immigration status): consult with safeguarding leads; risk-benefit articulation.
- Audio/video recording pathway: assume capacity unless assessed otherwise; use *process consent* (check/re-check during/after encounter); include a disruption stop-rule (cease/adjust recording if it degrades care). Provide accessible participant information (easy-read/video). See recent CA precedent in adjacent care contexts for feasibility and safeguards (e.g., palliative care communication with people with intellectual disabilities) (22).

Used in this way, the CPA audit and coaching tool can support wards in meeting culture-of-care commitments around relational safety, equality and avoiding harm, by making visible the small, everyday interactional moves that either pull practice toward least-restrictive, trauma- and autism-informed care or push it toward coercion and exclusion.

## 7 Worked mini-cases

We include three short, line-numbered staged scenes (triage, ward MDT, perinatal safeguarding). Each scene illustrates how a different practice can tilt a decision toward or away from restriction, followed by the alternative next move and a better documentation line. These can be used as templates for local coaching.

### 7.1 Mini-case 1: cancelling leave on a women’s ward (detained woman)

L1 Consultant: So, Sarah, you’d like to go ahead with your escorted leave this afternoon?

L2 Sarah: Yes. You said if I managed two days without self-harm I could have half an hour outside.

L3 Ward Nurse: There *was* that incident last night with the door.

L4 Sarah: I slammed it once after the phone call. I went straight to the lounge after.

L5 Consultant: It suggests you’re still quite unstable.

L6 Sarah: I was upset, but I calmed down. I told staff I was okay.

L7 Ward Nurse: We do have to be careful.

L8 Consultant: And with your history of trauma and “difficulties”, unstructured time off the ward is risky.

L9 Sarah: So I’m being punished for getting upset?

L10 Consultant: We’re not punishing you, but I think we should cancel leave for now and review next week.

L11 Sarah: Then what’s the point of me trying?

L12 Ward Nurse: We’ll write that we’ve held leave due to risk.

*Alternative next move.* (After L6/L7):

“OK, so last night you were distressed after the phone call, slammed the door once, then came to the lounge and settled. That’s important information. Let’s think how we can make today’s leave feel safe for you and for us. One option is a shorter escorted walk with a clear plan if you feel overwhelmed; another is to move the time so it doesn’t clash with calls. Which feels manageable, and what would you need from staff to make it safe?”

*Documentation rewrite.*

“Detained woman on ward requested escorted leave after two days without self-harm. One episode of distress the previous evening (slammed door once after difficult phone call; then joined lounge and settled with staff present). Discussed options; agreed 15-minute escorted walk at 15:00 with agreed signal to return early if overwhelmed. Trauma history and previous self-harm noted; plan balances safety with graded increase in autonomy. Review planned at evening handover.”

### 7.2 Mini-case 2: refusal → risk escalation on women’s acute ward (ward round)

L1 SHO: We’re proposing Q15 observations today.

L2 Patient: I don’t want people staring at me.

L3 Consultant: We need to observe you, it decreases risk.

L4 Patient: I’ll stay on the ward, just not checks every quarter hour.

L5 Nurse: Non-compliant yesterday as well.

L6 Patient: I asked to shower alone.

L7 Consultant: Given refusal, we’ll also cancel escorted leave.

L8 Patient: That’s not fair.

L9 Social Worker: She’s due to see her child later.

L10 Consultant: We need to set firm boundaries.

L11 Patient: Please—can we find another way?

L12 Nurse: We haven’t the time to argue about this now.

L13 Consultant: Decision stands.

L14 Patient: …

*Alternative next move*.

After L2: “We can meet the safety aim by (i) Q30 with proximity checks not eye-contact; (ii) patient-initiated check-ins every 20–30 min; (iii) temporary buddy system.” After L4: honor partial acceptance; trial for 2 hours with review.

*Documentation rewrite*.

“Detained woman on women’s acute ward expressed discomfort with visual checks; agreed to alternative: proximity checks + patient-initiated call button. Leave maintained for child contact with escort; review at 15:00. No incidents on trial period.”

### 7.3 Mini-case 3: category-bound inference in perinatal safeguarding (woman under secondary mental health care)

L1 Perinatal-Reg: Mood today?

L2 Mother: Tired; anxious about feeding.

L3 Safeguarding: Concerns about capacity as a mother remain.

L4 Mother: My partner is here full-time this week.

L5 HV: She missed two appointments.

L6 Mother: They clashed with midwife checks.

L7 Safeguarding: Non-engagement is a pattern.

L8 Perinatal-Reg: Diagnosis—does she accept it?

L9 Mother: I don’t like that word.

L10 HV: She minimizes symptoms.

L11 Mother: I’m trying my best.

L12 Safeguarding: We should escalate.

L13 Perinatal-Reg: Let’s pause. What support has worked?

L14 Partner: Night feeds rota helped.

L15 HV: The breastfeeding counsellor call helped.

L16 Perinatal-Reg: Then we continue those, add a home visit at times that don’t clash, and review in one week.

*Alternative next move (earlier).* (After L5/L7):

“Two missed HV slots clashed with midwife checks; let’s schedule at fixed times partner is present. Keep counsellor calls and night-rota; add one home visit focused on feeding technique.”

*Documentation rewrite*.

“Perinatal mood low; logistical clashes produced missed HV slots. Plan: maintain effective supports (partner rota, counsellor calls), schedule HV at non-clashing times, review in 7 days. No safeguarding escalation at this stage; concrete contingencies specified.”

### 7.4 Why this matters

Treating refusal as a stance to work with rather than a pure risk sign can preserve leave and dignity for detained women on acute wards. Checking logistics and supports before invoking “capacity” or “non-engagement” in perinatal cases can reframe the plan without immediate escalation. Across these settings, small changes in how women’s concerns are heard, formulated and documented can make the difference between graded support and restrictive, potentially traumatizing responses, and can interrupt the quiet reproduction of stigma and inequality in everyday decision-making, especially for women whose mental health, learning disability, autism, ethnicity or immigration status already place them at a disadvantage.

## 8 Reporting checklist for PA/CPA studies

This section offers a brief reporting checklist for PA/CPA, analogous in spirit to reporting standards such as CONSORT or COREQ, so that other teams can see what was done and how to replicate it. Reports should be transparent enough that a reader could, in principle, walk the same path on the same materials and reach comparable conclusions.

### 8.1 Make the data and context clear

State where the corpus came from (service, setting, jurisdiction), which activities the extracts belong to (e.g. triage calls, ward rounds, safeguarding meetings), how long the extracts are, and how they were redacted and stored. Indicate the governance route taken (public-domain vs service-evaluation/research approvals) and any specific considerations around sensitive categories (e.g. pregnancy, immigration status, violence).

### 8.2 Describe how the passage was worked through step-by-step in plain terms

Explain how the scene was recovered, which turns were selected to show the action, how category invocations and risk formulations were treated as *formulation-in-use*, what documentation effects were noted (how lines were likely to “travel”), and what alternative next moves and alternative documentation lines were formulated. Make it clear that meaning is taken as use, not as a freestanding property of terms.

### 8.3 Report the warrants and checks applied

Note how you used next-turn or third-position evidence, stability under extension (adding neighboring turns or cases), and any data sessions with colleagues or stakeholders to test candidate readings. Summarize what was learned from deviant cases (instances that did not fit the initial reading) and how these shaped the final perspicuous presentation of the case family.

## 9 Limitations and scope

No single lever fixes restrictive practices. Organizational constraints, staffing levels and external pressures can limit what alternatives are practicable at a given moment. Documentation is sensitive; redaction must preserve accountability while protecting identities. Some services will have only partial or uneven records, and access to recordings may be tightly governed. These are real constraints, and the protocol does not pretend otherwise.

Methodologically, our use of single cases and small case-families has limits but also distinctive strengths. Following Sacks, we treat cases as sites in which the production, maintenance and repair of norms and structures are visible in detail: how proposals, refusals, risk formulations and category invocations are handled, how they are taken up or resisted, and how they are laminated into the record. The point is not that a single case “represents” a whole population, but that, worked perspicuously, it can show how local conditions are put together in practice under which stigma becomes attached to a woman’s concerns, restrictive practices (such as cancelled leave, increased observations or escalation) become live and then expected options, and existing inequalities are quietly reproduced. Neighboring cases are then curated to form a family resemblance set, not a statistical sample, so that these patterns in the handling of talk and documentation can be seen across different women, wards and decision-points.

In line with case study work more broadly, we do not seek generalization by counting frequencies across a large N, but by analytical generalization from strategically chosen cases (23, 24). Context-dependent, concrete knowledge can be expert knowledge (24); a carefully selected case (for example, a “hard” case where the service is otherwise well-resourced and reflective) can test and sometimes undermine comfortable assumptions about how stigma and restriction arise. In that sense, perspicuous cases can have a critical function: they show that what might appear inevitable (“of course leave had to be cancelled here”) is in fact the contingent result of a series of sequential and categorical moves that could have gone otherwise.

These limits and commitments do not weaken the point of the protocol; they clarify it. We are not offering a magic bullet or a new variable to be added to existing models of stigma. We are offering a way to make visible the local levers—in talk, in documentation, in categorization—through which restrictive options and stigmatizing readings become live and then travel, so that teams can coach safer alternatives, audit how cases are written up, and re-formulate practice where it is driving avoidable restriction.

## 10 Contributions & use-cases

For researchers, the contribution is a reproducible, interview-free approach to the analysis of ordinary care that comes with its own reliability discipline and can be applied to documents or recordings. For services, the contribution is practical: a way to examine specific scenes, name alternatives that preserve alliance and safety, and build those alternatives into supervision and incident review. For policy and regulation, the protocol offers scene-level indicators for restrictive-practice risk and suggests how documentation templates might be adjusted so that credibility-first and alternative-offering moves are made accountable.

## References

[1] Women’s Health Strategy for England . GOVUK (2022). Available online at: https://www.gov.uk/government/publications/womens-health-strategy-for-england (Accessed January 1, 2026).

[2] NHS England . NHS England -- Culture of Care Standards for Mental Health Inpatient Services. Available online at: https://www.england.nhs.uk/long-read/culture-of-care-standards-for-mental-health-inpatient-services/ (Accessed December 11, 2025).

[3] Wittgenstein L . Philosophical Investigations. 4th ed. Hacker PMS Schulte J , editors. Oxford: Wiley-Blackwell (2009). p. 589.

[4] Ebersole FB . Things We Know: Fifteen Essays on Problems of Knowledge. 2nd ed. (Bloomington IN: Xlibris) (2001).

[5] Garfinkel H . Studies in ethnomethodology. Prentice-Hall., New York (1967).

[6] Sacks H . Lectures on Conversation. Vol. I. Jefferson G , editor. (Oxford: Blackwell) (1992). p. 818.

[7] Sacks H . Lectures on Conversation. Vol. II. Jefferson G , editor. (Oxford: Blackwell) (1992). p. 580.

[8] Long RT Jolley KD . Grammatical investigations. In: Jolley KD , editor. Wittgenstein: Key Concepts. (Oxford: Acumen Publishing) (2010). p. 169–74. doi: 10.1017/UPO9781844654420.015

[9] McGinn M . Grammar in the Philosophical Investigations. In: Kuusela O McGinn M , editors. The Oxford Handbook of Wittgenstein. (Oxford: Oxford University Press) (2011).

[10] Hutchinson P . Stigma and Shame. In: Handbook of the Philosophy of Medicine. (Dordrecht: Springer) (2025). p. 1635–54. doi: 10.1007/978-94-017-8706-2_93-1

[11] Hutchinson P . “Who is Watching Me:” Recovering the Grammar of Stigma in Interaction. (2026).

[12] Hardman D Hutchinson P . Praxeological analysis: A new qualitative methodology. Int J Qual Methods. (2025) 24:16094069251333894. doi: 10.1177/16094069251333894

[13] Diskin K Hutchinson P . Critical praxeological analysis: respecifying critical research. Qual Res Psychol. (2024) 21:512–35. doi: 10.1080/14780887.2024.2365862

[14] Schegloff EA Sacks H . Opening up closings. Semiotica. (1973) 8:289–327. doi: 10.1515/semi.1973.8.4.289

[15] Schegloff EA . The Adjacency Pair as the Unit for Sequence Construction. In: Sequence Organization in Interaction: A Primer in Conversation Analysis. (Cambridge: Cambridge University Press) (2007). p. 13–21. doi: 10.1017/CBO9780511791208.003

[16] Sacks H . On the Analysability of Stories by Children. In: Gumperz JJ Hymes D , editors. Directions in Sociolinguistics. (Oxford: Blackwell) (1972). p. 325–45.

[17] Stokoe E . Moving forward with membership categorization analysis: methods for systematic analysis. Discourse Stud. (2012) 14:277–303. doi: 10.1177/1461445612441534

[18] Smith RJ . Membership Categorisation Analysis. In: The Routledge International Handbook of Ethnomethodology. (London: Routledge) (2025).

[19] Hutchinson P . Stigma respecified: investigating HIV stigma as an interactional phenomenon. J Eval Clin Pract. (2022) 28:861–6. doi: 10.1111/jep.13724, PMID: 35709243 PMC9796448

[20] Bauer MW Gaskell G . Qualitative Researching with Text, Image and Sound. (London: SAGE Publications Ltd) (2000).

[21] NHS England . NHS England -- Patient and Carer Race Equality Framework (2023). Available online at: https://www.england.nhs.uk/publication/patient-and-carer-race-equality-framework/ (Accessed December 11, 2025).

[22] Bruun A Tuffrey-Wijne I . Palliative care communication between patients with intellectual disabilities and hospice staff: a conversation analysis pilot study protocol. BMJ Open. (2025) 15:e101622. doi: 10.1136/bmjopen-2025-101622, PMID: 40578897 PMC12207180

[23] Flyvbjerg B . Case Study. In: Denzin NK Lincoln YS , editors. Sage Handbook of Qualitative Research. (Rochester, NY: Sage) (2011). 301–16.

[24] Flyvbjerg B . Five misunderstandings about case-study research. Qual Inq. (2006) 12:219–45. doi: 10.1177/1077800405284363

